# Otoferlin gene editing in sheep via CRISPR-assisted ssODN-mediated Homology Directed Repair

**DOI:** 10.1038/s41598-020-62879-y

**Published:** 2020-04-07

**Authors:** A. Menchaca, P. C. dos Santos-Neto, M. Souza-Neves, F. Cuadro, A. P. Mulet, L. Tesson, V. Chenouard, A. Guiffès, J. M. Heslan, M. Gantier, I. Anegón, M. Crispo

**Affiliations:** 1 Instituto de Reproducción Animal Uruguay, Fundación IRAUy, Montevideo, Uruguay; 2 Unidad de Animales Transgénicos y de Experimentación (UATE), Institut Pasteur de Montevideo, Montevideo, Uruguay; 3 Inserm, Centre de Recherche en Transplantation et Immunologie, UMR 1064, F-44000 Nantes, France; 4 Transgenesis Rat ImmunoPhenomic facility (TRIP), F-44000 Nantes, France; 5 GenoCellEdit facility, F-44000 Nantes, France

**Keywords:** Genetic engineering, Experimental models of disease

## Abstract

Different mutations of the *OTOF* gene, encoding for otoferlin protein expressed in the cochlear inner hair cells, induces a form of deafness that is the major cause of nonsyndromic recessive auditory neuropathy spectrum disorder in humans. We report the generation of the first large animal model of *OTOF* mutations using the CRISPR system associated with different Cas9 components (mRNA or protein) assisted by single strand oligodeoxynucleotides (ssODN) to induce homology-directed repair (HDR). Zygote microinjection was performed with two sgRNA targeting exon 5 and 6 associated to Cas9 mRNA or protein (RNP) at different concentrations in a mix with an ssODN template targeting HDR in exon 5 containing two STOP sequences. A total of 73 lambs were born, 13 showing indel mutations (17.8%), 8 of which (61.5%) had knock-in mutations by HDR. Higher concentrations of Cas9-RNP induced targeted mutations more effectively, but negatively affected embryo survival and pregnancy rate. This study reports by the first time the generation of *OTOF* disrupted sheep, which may allow better understanding and development of new therapies for human deafness related to genetic disorders. These results support the use of CRISPR/Cas system assisted by ssODN as an effective tool for gene editing in livestock.

## Introduction

Since the first report of CRISPR edited mammals was published in 2013 in mice^1^, the CRISPR/Cas system has revolutionized the field of genome editing for a number of species. The first birth of knockout (KO) sheep in our laboratory occured one year later in 2014^2^ with available livestock models increasing rapidly during the last years. As uses of CRISPR/Cas9 have proliferated, its uses have broadened to include enhanced traits production^2^, disease resistance^3^, xenotransplantation^4^, and also the study of human diseases^5,6^. In most of the reports published in livestock^7^ CRISPR/Cas9 is generally used to create a double-strand break, which induces the non-homologous end joining (NHEJ) repair pathway disrupting functional alleles. The resulting deletions and insertions lead to a functional KO of the targeted gene^8^. Although CRISPR/Cas9 system can also trigger the homology directed repair (HDR) pathway, reports employing this strategy to mediate the knock-in (KI) of exogenous donor DNA have generally been limited to mice or rats, with little success in livestock^9^. Another promising approach that requires further study in livestock is to use single-stranded oligodinucleotides (ssODNs) to induce HDR of DNA by single-strand annealing^8^.

One application of genome editing in large species is the design of animal models to better understand human diseases and development of new therapies, something that was difficult to achieve before the development of the CRISPR tool. Hearing loss is the most common neurosensory disorder in humans, which can be a congenital pathology related to one or several genes^10^. According to the last update of the World Health Organization (WHO), approximately 466 million people worldwide suffer from a disabling hearing loss (https://www.who.int/deafness/en/). This disease is described as an etiologically heterogeneous pathology caused by different genetic and environmental factors, with approximately half of the cases being genetic^11^. The *OTOF* gene, which encodes for otoferlin protein, is mostly expressed in the cochlear inner hair cells, and is necessary for synaptic exocytosis at the auditory ribbon synapse, as previously shown in OTOF KO mice^12^. The first report of otoferlin protein appeared in 1999, when it was discovered that *OTOF* gene mutations cause a nonsyndromic form of deafness^13^. Moreover, certain mutations in the *OTOF* gene are reported to be the major cause of nonsyndromic recessive auditory neuropathy spectrum disorder in humans^14^. Studying these mutations in a large animal model could provide a better understanding of the role of *OTOF* and to further test different therapies and possibly rescue the function of hair cells. To our knowledge, no reports about the use of large animal models to study this kind of deafness and its possible therapies exist.

Although promising, modifying gene expression through insertion of short DNA sequences in large animals has rarely been reported. In addition, most of the reports using CRISPR-Cas system in large animals (by NHEJ), have been based mainly in the use of *in vitro* transcribed Cas9 mRNA. Recently, using Cas9 as a ribonucleotide particle (RNP) has gained popularity in rodents and cell culture due to the greater nuclease activity of Cas9 protein when compared to Cas9 mRNA, as reported in rat^15^ and mouse zygotes, as well as in somatic cells^16^. However, comparisons of efficiency of both types of Cas9 components remain scarce in many species, and to our knowledge no such report has been published in ruminants (*i*.*e*., sheep, goats and cattle) or other large mammals. Thus, improving the targeted integration efficiency remains a challenge in livestock because using Cas9 protein in this way is an approach still in development.

The current study reports for the first time the use of the CRISPR/Cas9 system to develop a KI sheep model using ssODN to induce two stop codons to produce a KO for *OTOF* in exon 5 or 6. We compare the efficiency of both the Cas9 mRNA or RNP strategies.

## Results

### *In vitro* efficiency assessment in embryos

To determine the best mRNA or RNP concentrations to obtain the KI sheep, ovine zygotes were microinjected, cultured to the blastocyst stage and genotyped. Capillary electrophoresis performed in embryos showed heteroduplexes in many blastocyst samples due to either NHEJ reparation after CRISPR-mediated DNA cleavage or to different SNPs present in different embryos (data not shown). Sequence analysis of the PCR products allowed to define which ones were mutated by NHEJ or were just potential SNPs present in different animals **(**Supplementary Table S1**)**. From 51 blastocysts analyzed, sgRNA targeting OTOF exon 5 had better efficiency than sgRNA targeting exon 6 (10 vs. 4 blastocysts, respectively). Two embryos had both exons mutated. For both exon 5 and 6, repair by microhomology NHEJ led to frequent deletions of multiples of 3 nt, without open reading frame shifts but rather deletion of a few amino acids. Overall, there were at least 4 KO embryos and potentially more in the embryos that showed multiple deletions which were not precisely defined. Frequency of NHEJ for exons 5, 6 or both was as follow: the efficiency of Cas9 RNP at 500 ng/µl was 25.0% (5/20), the efficiency of Cas9 RNP at 50 ng/µl was 5.3% (1/19), and the efficiency of Cas9 mRNA at 50 ng/µl was 50.0% (6/12) with two of the mutated embryos showing both exons mutated. A significant difference in the KO efficiency was found for Cas9 mRNA at 50 ng/µl (*P* < 0.05). Using these results, we decided to generate the KI sheep by injecting the zygotes using either Cas9 mRNA or Cas9 RNP in concentrations higher than 50 ng/ul.

### *In vivo* mutation efficiency in lambs

The efficiency obtained for the different CRISPR/Cas9 systems is summarized in Table 1. From a total of 247 recipients, 62 were pregnant 30 days after embryo transfer. Regarding the pregnancy rate for each microinjection condition, the highest efficiency (45.8%, pregnant/transferred ewes) was obtained with the lowest Cas9 protein concentration (RNP 100 ng/µl). The pregnancy rate for the others microinjection conditions was lower (*P* < 0.05), ranging from 16.1% for Cas9 mRNA to 24.5% and 23.1% for Cas9 RNP 250 and 500 ng/µl, respectively.

**Table 1:** Efficiency of *OTOF* mutant lambs generation induced by CRISPR-Cas9 system using either Cas9 mRNA or Cas9 protein (RNP).

	Overall results	Cas9 mRNA *vs*. Cas9 RNP				
		mRNA 50 ng/µl	RNP 100 ng/µl	RNP 250 ng/µl	RNP 500 ng/µl	*P* value
Number of recipient females	247	103	48	49	47	—
Number of transferred embryos	1,316	629	218	236	233	—
Pregnant/transferred recipients	25.1% (62/247)	16.5%^a^ (17/103)	45.8%^b^ (22/48)	24.5%^a^ (12/49)	23.4%^a^(11/47)	<0.05
Embryos alive at 30 d of gestation	78	19	33	14	12	—
Fetal losses (from 30 d of gestation to birth)	6.4% (5/78)	5.3% (1/19)	6.1% (2/33)	7.1% (1/14)	8.3% (1/12)	NS
Lambs born	73	18	31	13	11	—
Lamb survival rate*	89.0% (65/73)	77.8% (14/18)	93.5% (29/31)	100% (13/13)	81.8% (9/11)	NS
Mutants/lambs born	17.8% (13/73)	27.8%^a^ (5/18)	6.5%^b^ (2/31)	7.7%^b^ (1/13)	45.5%^a^ (5/11)	<0.05
KI/mutant lambs	61.5% (8/13)	60.0% (3/5)	50.0% (1/2)	100% (1/1)	60.0% (3**/5)	NS
KI/total lambs	11.0% (8/73)	16.7% (3/18)	3.2% (1/31)	7.7% (1/13)	2.7% (3**/11)	NS

For superscripts a vs. b, p < 0.05.

*Lambs alive at first week after birth.

**Out of three lambs, two are mosaic.

**Figure 1:**
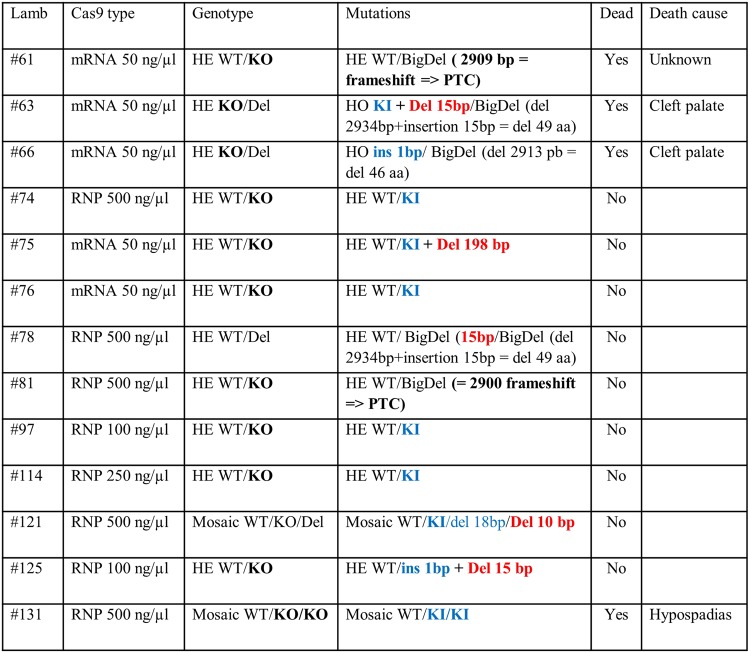
Genotypes of produced lambs for *OTOF* mutations using the CRISPR/Cas9 technology. Blue color, exon 5; Red color, exon 6; HO, homozygous; HE, heterozygous; slash (/), separates the genotypes of the two chromosomes; WT, wild-type; KO, knockout; Del, deletion; KI, insertion of STOP codons; PTC, premature termination codon due to NHEJ leading to a KO. In bold all the mutations that induce a KO. All DNA samples were extracted from skin except #63 and #66 (skin and muscle), #97 (skin, muscle and blood).

Fetal losses were 6.4%, as determined by number of embryos lost between pregnancy diagnosis and lambs born at delivery (5/78), which was unaffected by microinjection conditions. At delivery, a total of 73 lambs were born, some of them twins. All lambs were biopsied, and DNA was extracted and genotyped using the technique described in the Methods. Lamb survival rate was not affected by microinjection conditions (P = NS, Table 1) and was in average 89.0% (65/73). Low sample size notwithstanding, lamb survival rate within one week after birth was greater in WT (56/60, 93.3%) than in mutant lambs (9/13, 69.2%; P < 0.05). Of the 8 lambs dead at delivery or soon thereafter, some of them showed cleft palate (two lambs, both mutants, #63 and #66), brachycephaly (2 lambs, both WT), hypospadias (1 mutant lamb, #131) and the others dead from unknown causes (1 mutant, #61; and 2 WT) (mutant lambs are shown in Fig. 1**)**.

Overall mutant rate in lambs was 17.8% (13/73, mutants/lambs born) and the rest of the animals (60/73) revealed a WT genotype. Those 13 lambs that presented an edited genotype are presented in Fig. 1. KI with the ssODNS containing the STOP signals was successful in 61.5% of edited lambs (8/13), or 11% of the total lambs (8/73) (Table 1). Of these 13 lambs, 8 showed a heterozygous genotype (WT/KO; #61, 74, 75, 76, 81, 97, 114 and 125) with a KO allele (either via KI of the ssODN with stop signals, or indels generating a premature stop codon (PTC)) and a WT allele. Of these 8 lambs, 5 had a KO allele via ssODN KI of the STOP signals (#74, 75, 76, 97, 114), 1 had a KO allele due to one bp insertion in exon 5 (#125), and 2 had large deletions (#61 and 81, with 2909 bp and 2900 bp, respectively) between exon 5 and 6 with frameshifts leading to PTCs.

**Figure 2:**
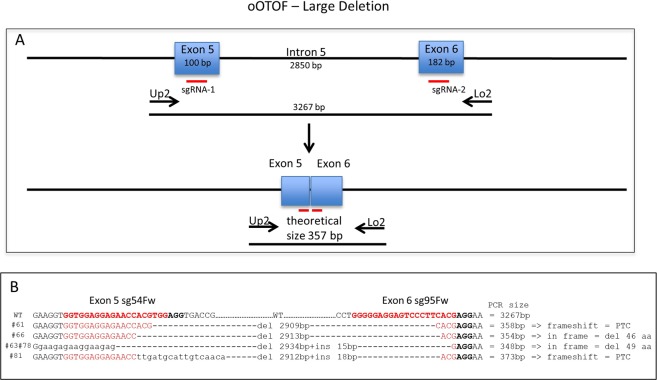
Characterization of large deletions between exon 5 and 6. Panel a) PCR scheme with primers situated in 5′ of exon 5 and in 3′ of exon 6 revealing a band of 3267 bp for a WT or a theoretical 357 bp band in case of double DNA cut and deletion without indels. The location of the two sgRNA used are indicated (red bars). Panel b) For 5 lambs with deletions, the sequence of these PCRs showed variable deletions with insertion (#61, #63 and #78) or not (#61 and #66) depending on microhomologies. The sequences recognized by the two sgRNA used are indicated (red letters) and the PAMs in black bold. Between the sequences the size of the deletions and of the insertions and at the right hand side the real PCR size for each lamb with the final outcomes. PTC: Premature Termination Codon.

The other 5 mutated lambs showed a more complex genotype **(**Fig. 1**)**. Three of them showed large deletions (#63, 66 and 78, with 15 bp, 2913 bp and 2919 bp, respectively) without frameshift. Only 1 of them (#78) had a WT/Del genotype. Lamb #63 had two alleles with deletions, one allele with a KI in exon 5 plus a 15 bp deletion in exon 6 and the other allele with a deletion of 15 bp between exon 5 and 6 without frameshift. Lamb #66 had one KO allele due to one bp insertion in exon 5 and one allele with a 2913 bp deletion between exon 5 and 6 without frameshift **(**Fig. 2**)**. The other 2 lambs with complex genotypes (#121, 131) were determined to be mosaics because they presented at least 3 alleles (WT/KO/Del).

**Figure 3:**
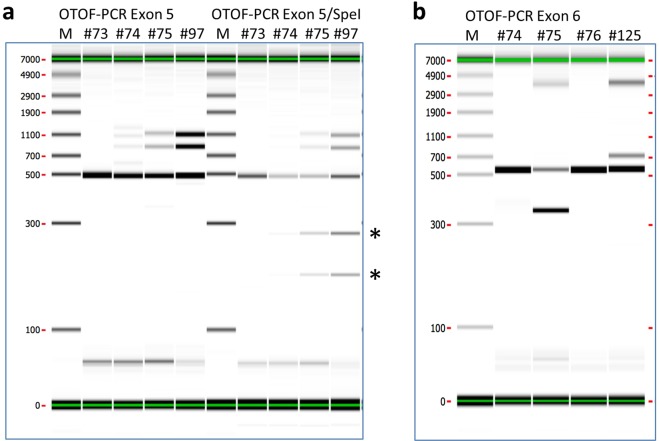
oOTOF gene knock-out/knock-in exon 5 and 6. Panel a) Example of typical result for exon 5 PCR (left lines) and Spe I exon 5 PCR digest (right lines). Main band is around 500 bp. The bands above the main band in all animals except #73 (WT/WT) are heteroduplexes which means that NHEJ and/or KI occurred. Spe I digest revealed two bands (asterisk) under the main band of animals #74, #75 and #97 and means that a KI occurr. Panel b) Example of typical result for exon 6 PCR. Main band is around 500 bp. The bands above the main band in all animaedls except #74 and #76 (WT/WT) are heteroduplexes which means NHEJ occurred. Animal #75 shows a smaller band of ~400 bp under the main band which identifies a 198 bp deletion.

Examples of the different genotypes are presented in Fig. 3. In exon 5, except #73 which is a WT, the lambs #74, 75, 97 showed heteroduplexes which indicate that NHEJ and/or KI occurred. To confirm KI, a SpeI digest revealed two bands under the main band of animals #74, 75, 97. In exon 6, except #74 and 76, animal #75 and 125 showed heteroduplexes which indicates NHEJ occurred and #75 showed a smaller band which signed a 198 bp deletion.

Analysis of four potential off-target sites situated in exons (described in Supplementary Table S2) for each sgRNA targeting exon 5 and 6 (8 sites per animal) were analyzed by PCR followed by T7 endonuclease digestion and capillary electrophoresis. Analysis of 13 mutated lambs and 2 WT animals showed an absence of indels in all 8 potential off target sites (data not shown).

Overall, from the 13 edited lambs, 5 were obtained with mRNA and 8 were obtained with RNP. None of these lambs showed a clear homozygous KO genotype.

## Discussion

We developed mutant KO lambs for the *OTOF* gene using the CRISPR/Cas9 system, combining ssODNs and different Cas9 conditions in order to improve mutation efficiency. This is the first report of a KO *OTOF* model in large animals, which could be useful to better understand of deafness in humans as well as to test different therapies to treat this genetic disorder. As one of the early large animal models generated through the CRISPR/Cas system, this study provides novel information for future applications of this technology in livestock.

Selecting the optimal approach for genome editing and embryo transfer are critical because time and costs of conducting these kind of projects in large animals represent a limiting factor. We thus suggest that *in vitro* efficiency assessment in embryos is a recommended practice to generate CRISPR-mutated farm animals. *In vitro* efficiency assessment in embryos showed that mRNA at 50 ng/µl and RNP at 500 ng/µl appear to be feasible approaches to obtain *OTOF* mutant embryos (50% and 25%, respectively; *P* = NS). This information was used to define the microinjection conditions for embryo transfer and generation of the mutant model. The lowest concentration of Cas9 protein (*i*.*e*., 50 ng/µl) was not effective and allowed us to move towards the generation of the model by microinjection of Cas9 mRNA and higher RNP concentrations.

Overall mutant rate in lambs was 17.8% (13/73, mutants/lambs born) and 8 of 13 had the KI mutation, representing 61.5% efficiency. The highest efficiency was obtained when Cas9 RNP was injected at 500 ng/µl, with almost 50% of the lambs born showing mutations in the desired region, providing a high efficiency for this RNP concentration. Lower RNP concentrations (100 or 250 ng/µl) generated a lower mutation rate as expected, although pregnancy rate was higher with the lowest RNP concentration (100 ng/µl). Our results show that as the amount of microinjected RNP increases, proportion of mutants increases but pregnancy rate falls.

The use of Cas9 mRNA instead of protein allows acceptable mutation efficiency in lambs, which was intermediate between 250 ng/µl and 500 ng/µl of RNP. Although both Cas9 mRNA at 50 ng/µl and Cas9 RNP at 500 ng/µl seem to be detrimental to embryo and lamb survival rate, surviving lambs showed a higher mutation rates. In addition, the concentration of Cas9 protein increased along with the concentration of sgRNAs and ssODN, possibly affecting the efficiency obtained with mRNA versus protein injection. These outcomes indicate that reagent concentrations should be further optimized under *in vitro* evaluation for each gene editing project to find the best balance between CRISPR efficiency and embryo survival. Our results are in agreement with findings in human cell culture experiments^16^, which report that RNP at higher concentration are the most convenient and efficient approach to use to maximize mutant rates. However, when working in embryos and livestock species, embryo survival and pregnancy rate, in addition to mutant rate, are also key factors for the project’s success.

In order to maximize the efficiency to obtain the *OTOF* KO model not depending only on NHEJ repair mechanism introducing indels, an ssODN of 108 bp with small homology arms was designed to introduce two premature stop codons. With this approach we achieved KO in 17.8% of lambs, 61.5% of which contained the KI, demonstrating that this strategy was effective to produce this mutant model with high HDR rates. Our data confirms the relevance of including an ssODN template to more precisely induce in the desired mutation. ssODN are one of the main types of homology repair templates used for making small genetic changes (about 50–100nt). The high fidelity of the homologous recombination pathway can be used to create controlled insertions, deletions and substitution of a single nucleotide or large tracks of genomic DNA by HDR. Although reported in other species^17–19^, current literature is scarce for CRISPR assisted ssODN-mediated homologous recombination in ruminant species (*i*.*e*., sheep, goats and cattle). Recently, Williams *et al*.^5^ reported for the first time the effectiveness of this tool to induce a point mutation in sheep, and Eaton *et al*.^20^ described the generation of homozygotes lambs by the insertion of a human mutation into the orthologous sheep locus. In the current study, we successfully generated both indel mutations and HDR-mediated KI using the ssODN strategy in sheep. The inclusion of ssODNs was an effective tool to achieve an acceptable mutant efficiency by HDR-based gene editing method in this species. Although the long range PCRs used in this study and the genotyping using capillary electrophoresis followed by amplicon sequencing allow quite precise genotyping^21^, the use of next generation sequencing would have allowed a more precise genotyping of mosaic animals

The present report shows the efficient generation of the first large animal model with *OTOF* null mutations, which would be useful for the understanding of otoferlin function and human deafness. Different mutations in the *OTOF* gene lead to severe deafness in humans, since otoferlin is involved in vesicle membrane fusion and its expression in the sensory hair cells is linked to synaptic vesicles^14^. We characterized the genotype of this model in sheep, a species more appropriate than murine models to study the auditory system due to similar human cochlear dimensions and anatomy. Future work may include crossing heterozygous male and female *OTOF* KO animals to obtain homozygous *OTOF* KO lambs which will be phenotyped to confirm hearing loss. Deaf homozygous *OTOF* KO animals will allow further phenotypic characterization of the model to analyze the congenital disease and to test different therapies in the field of deafness.

In conclusion, we have successfully developed the first reported CRISPR sheep model carrying different mutations resulting in abrogation of *OTOF* expression, opening the door for basic and applied research in hearing impairment. The sheep model allows a better balance than mice regarding model size for the study of human diseases and therapies. The strategy designed for this study, consisting of two sgRNA, different Cas9 mRNA and protein concentrations, and the addition of ssODN templates, showed an acceptable efficiency and adds novel information to the nascent literature on livestock gene editing models generated by CRISPR/Cas system.

## Methods

### Ethical approval

All applicable international, national, and/or institutional guidelines for the care and use of animals were followed. All procedures performed in studies involving animals were in accordance with the ethical standards of the institution (Fundación IRAUy). The procedures were approved by the Internal Ethical Committee for the Use of Animals of IRAUy (CEUA-IRAUy protocol #001-2017).

### Generation of sgRNA and ssODN to produce *OTOF* deficient sheep

**Figure 4:**
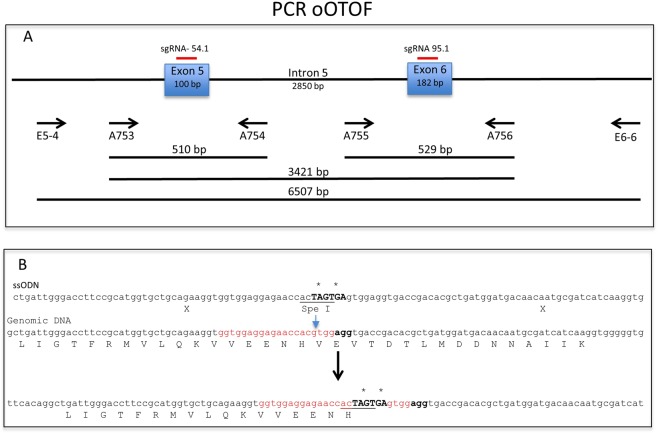
oOTOF gene knock-out strategy. Panel a) Two sgRNA were designed to target exon 5 and exon 6 (sgRNA 54.1 and 95.1, respectively). Below, arrows mark the positions of the primers described in Supplementary Table 2 that were used for genotyping and lines the size of the different PCR products. Primers were designed to amplify each target zone to define if KO/KI happened in exon 5 or KO in exon 6. Large PCR were done to analyse if large deletion occured between exon 5 and 6. Panel b) A ssODN (108 bp) with short homology arms (51 bp each arm) was designed to introduce in exon 5 two premature stop codons (bold, back, marked with asterisks) and a restriction site (SpeI, underlined) to facilitate genotyping. Blue arrow is cleavage site of the genomic DNA by Cas9, 3 bp upstream of the PAM, AGG in lower case, bold). The restriction site insertion one base upstream of the cleavage site was designed to use the AC sequence to generate a SpeI site (ACTAGT) by the introduction of the two stop codons TAGTGA. In red, sequence recognized by sgRNA 54.1 on exon 5. The lower diagram shows the sequence after introduction of the two stop codons immediately upstream of the cleavage site. This sequences will alter transcription and will not be recognized by sgRNA 54.1.

Two sgRNA were designed and produced by the GenoCellEdit facility (Nantes, France) using CRISPOR software. Both sgRNA 54.1 and 95.1 were used to target exon 5 and exon 6 of *OTOF* gene, respectively, and have the following sequence: GGTGGAGGAGAACCACGTGG for exon 5 and GGGGGAGGAGTCCCTTCACG for exon 6. Each sgRNA was *in vitro* tested by electroporation in A15 sheep cells as described^2^. Efficiencies were assessed after a T7 endonuclease I test followed by capillary electrophoresis, and calculated as a ratio between cut and uncut band **(**Supplementary Fig. S1**)**. In order to maximize the editing efficiency for the *OTOF* KO model not depending only on NHEJ repair mechanism introducing indels, a ssODN of 108 bp with short homology arms was designed to introduce two premature stop codons as well as a restriction site (Spe I) to facilitate the genotyping **(**Fig. 4**)**. With the introduction of these mutations, the sequence recognized by the sgRNA 54.1 is eliminated.

### Embryo production for microinjection

One-cell embryos for microinjection were obtained using standard procedures as previously described in our laboratory^22^. Briefly, cumulus oocytes complexes (COCs) were retrieved from slaughterhouse ovaries of wild-type sheep. Grade A and B COCs were matured *in vitro* in maturation media for 24 h in 5% CO_2_ at 39 °C in humidified atmosphere. Insemination of matured COCs was performed with 1 ×10^6^ dose/100 ul drop of insemination media using frozen-thawed semen previously collected from a wild-type ram and subjected to sperm selection by swim-up. Presumptive zygotes were denuded 16 to 18 hours post insemination by gently pipetting to prepare them for microinjection.

### *In vitro* efficiency assessment in embryos

To determine the best mRNA or RNP concentrations to obtain the sheep model, ovine zygotes were microinjected with a mix of the two OTOF sgRNAs targeting exon 5 and 6, using either Cas9 mRNA (50 ng/µl) (Invitrogen, CA, USA) or Cas9 RNP (Synthego, CA, USA) at two different concentrations (50 or 500 ng/µl). Zygote microinjection was performed into the cytoplasm as previously described^2^. Each microinjection mix was back loaded into homemade injection glass capillaries that were immediately introduced into the cytoplasm of individual zygotes fixed by a holding pipette. Approximately 2 to 5 pl of the microinjection mix were injected into each zygote, using the Eppendorf system Transferman NK2 micromanipulators and Femtojet pressure control device (Eppendorf, Hamburg, Germany). After microinjection, surviving zygotes were left in culture (SOF medium supplemented with 5% BME essential amino acids, 2.5% MEM nonessential amino acids and 0.4% BSA) until the blastocyst stage. DNA from individual blastocysts from each group was extracted using Arcturus PicoPure DNA Extraction kit (Thermo Fisher Scientific, MA, US) and analyzed by capillary electrophoresis and Sanger sequencing.

### Zygote microinjection of CRISPR/Cas system for embryo transfer

Different conditions for microinjection were selected from the above experiment: (a) Cas9 mRNA (50 ng/µl) mixed with sgRNA guides (10 ng/µl each guide) and ssODN (20 ng/µl) in standard microinjection buffer (10 mM Tris pH 7.5, 0.1 mM EDTA); or (b) Cas9 RNP (100, 250 or 500 ng/µl) mixed with sgRNA guides (40, 100 or 200 ng/µl each guide, respectively) and ssODN (40, 100 or 200 ng/µl, respectively) in protein microinjection buffer (20 mM hepes, 150 mM KCL). When using Cas9 protein, the mix was incubated at room temperature for 10 min to allow the formation of RNP complexes and then maintained at 4 °C until microinjection performed (within 1 h). Microinjection was performed as described above and surviving embryos were left in culture until transfer or vitrification.

### Embryo transfer and pregnancy diagnosis

A total of 1,316 microinjected embryos with Cas9 mRNA (n = 629) and Cas9 RNP (n = 687) were transferred fresh or vitrified within six days after *in vitro* fertilization into 247 recipient ewes (n = 103 and 144 for Cas9 mRNA and Cas9 RNP, respectively) previously subjected to estrous synchronization. The embryo transfer procedure was assisted by laparoscopy (Karl Storz, Tuttlingen, Germany) and pregnancy diagnosis was performed 30 days after *in vitro* fertilization using transrectal B-mode ultrasonography equipped with a 5 MHz probe (Well-D, Shenzhen, China).

The recipient ewes were maintained in typical pasture conditions with *ad libitum* access to food and water. All surgeries were performed under strict aseptic conditions, and all efforts were made to minimize animal suffering. In total, 73 lambs were produced and genotyped for mutations.

### Genotyping of lambs

Samples of skin, muscle or blood were retrieved one week after lamb’s delivery. DNA extraction was performed using a standard protocol from our laboratory. Briefly, skin or muscle samples were digested with Proteinase K (Eurobio, France) in lysis buffer (10 mM Tris pH 8, 100 mM NaCl, 10 mM EDTA pH 8, 0.5% SDS) to obtain total DNA. Erythrocytes in blood samples were lysed using 10 mM Tris PH 7.4, 320 mM sucrose, 5 mM MgCl and 1% Triton 100x prior to digestion of the remaining blood cells. The CRISPRs nuclease targeted region was PCR amplified from purified gDNA (50 ng) with a high-fidelity polymerase (Herculase, Agilent, CA, USA). To detect gene editing or the KI mutation, specific oOTOF primers were used (see Supplementary Table S3). Mutations were analyzed by capillary electrophoresis (Caliper, PerkinElmer, Hopkinton, MA) using a technique previously described in detail^21^, Spe I digestion for oOTOF exon 5 KI and direct sequencing of PCR products. To detect potential off target effects, we analyzed the four most homologous genomic sequences recognized by the sgRNAs used to mutate exon 5 and 6 (as determined by the CRISPOR software and by Doench *et al*.^23^). This analysis was performed by PCR and amplicon Sanger sequencing.

### Statistical analysis

Generalized linear mixed models (GLMM) were performed using Infostat software^24^ to compare different variables among experimental groups, with the treatment as fixed effect and the replicate as random effect. The significance level was established in a p value of 0.05.

## Supplementary information

Supplementary information.
