# Supplementary material for: Otoferlin gene editing in sheep via CRISPR-assisted ssODN-mediated Homology Directed Repair
Source: Sci Rep. 2020 Apr 7;10:5995. doi: 10.1038/s41598-020-62879-y (PMC7138848; doi:10.1038/s41598-020-62879-y)
Supplement: Supplementary file 1 — Supplementary information. [file 41598_2020_62879_MOESM1_ESM.docx]

**Otoferlin gene editing in sheep via CRISPR-assisted ssODN-mediated Homology Directed Repair**

Menchaca A^1*^, dos Santos-Neto PC^1^, Souza M^1^, Cuadro F^1^, Mulet AP^2^, Tesson L^3,4^, Chenouard V^3,4^, Guiffès A^3,4^, Heslan JM^3,5^, Gantier M^3,5^, Anegón I^3,4,5*^, Crispo M^2*^

*^1^Instituto de Reproducción Animal Uruguay (IRAUy), Montevideo, Uruguay.*

*^2^Unidad de Animales Transgénicos y de Experimentación (UATE), Institut Pasteur de Montevideo, Uruguay.*

*^3^Inserm, Centre de Recherche en Transplantation et Immunologie,  UMR 1064, F-44000 Nantes, France.*

*^4^Transgenesis Rat ImmunoPhenomic facility (TRIP), F-44000 Nantes, France.*

*^5^ GenoCellEdit facility, F-44000 Nantes, France.*

***Corresponding authors**

[menchaca.alejo@gmail.com](mailto:Menchaca.alejo@gmail.com)

[ianegon@nantes.inserm.fr](mailto:ianegon@nantes.inserm.fr)

crispo@pasteur.edu.uy

Supplementary Table S1. *In vitro* genotype assessment in embryos after zygote microinjection of different conditions of Cas9 preparation (mRNA and protein).

| Cas9 mRNA 50 ng/µl | | | Cas9 RNP 50 ng/µl | | | Cas9 RNP 500 ng/µl | | |
| --- | --- | --- | --- | --- | --- | --- | --- | --- |
| Sample  (#embryo) | Exon 5 | Exon 6 | Sample  (#embryo) | Exon 5 | Exon 6 | Sample  (#embryo) | Exon 5 | Exon 6 |
| #1 | WT | Del 3 bp Ho | #1 | WT | WT | #1 | WT | WT |
| #2 | WT | WT/Del nd | #2 | WT | WT | #2 | WT | WT |
| #3 | WT | WT | #3 | WT | WT | #3 | WT | WT |
| #4 | WT | WT | #4 | WT | WT | #4 | WT/ Del nd | WT |
| #5 | WT | WT | #5 | WT | WT | #5 | WT/Del 15 bp | WT |
| #6 | Del 15 bp Ho | WT/ins 2 bp **KO** | #6 | WT | WT | #6 | WT | WT |
| #7 | WT/Del 15 bp | WT/Del nd | #7 | WT | WT | #7 | WT/Del 6 bp | WT |
| #8 | WT | WT | #8 | WT | WT | #8 | WT | WT |
| #9 | WT | WT | #9 | WT | WT | #9 | WT/Del 2 bp **KO** | WT |
| #10 | WT/ins 1 bp **KO** | WT | #10 | WT | WT | #10 | WT/Del 2 bp **KO** | WT |
| #11 | Del 2 bp/ins 1bp | WT | #11 | WT/ins 1 bp **KO** | WT | #11 | WT | WT |
| #12 | No data | WT | #12 | WT | WT | #12 | WT | WT |
|  |  |  | #13 | WT | WT | #13 | WT | WT |
|  |  |  | #14 | WT | WT | #14 | WT | WT |
|  |  |  | #15 | WT | WT | #15 | WT | WT |
|  |  |  | #16 | WT | WT | #16 | WT | WT |
|  |  |  | #17 | WT | WT | #17 | WT | WT |
|  |  |  | #18 | WT | WT | #18 | WT | WT |
|  |  |  | #19 | WT | WT | #19 | WT | WT |
|  |  |  |  |  |  | #20 | WT | WT |

nd (not defined): these embryos showed multiple deletions in which bp were not precisely defined.

Supplementary Table S2. List of potential off-target exons analyzed and the sequence of the primers used for their analysis.

| **oOTOF-Exon 5** |  |  |  |  |
| --- | --- | --- | --- | --- |
| **Primer Name** | **Primer Sequence** | **On-target sequence** | **Number of mismatches:Location/Chromosome** | **CFD Off-target score** |
| OTOF-Ex5-A753 | CACACAGTTGTTGGGGGTCA | GGTGGAGGAGAACCACGTGG AGG | 0:exon:OTOF/chr3 | 0 |
| OTOF-Ex5-A754 | CTCCTCCCACCGTACCTTTC |  |  |  |
|  |  | **Potential off-target sequence** |  |  |
| OT1-For | GAACAGCAGCAACAGGATGGA | GG**A**GGAGGAGAACCA**A**G**A**GG GGG | 3:intergenic:ENSOARG00000005814-SORBS1/chr22 | 0.48 |
| OT1-Rev | CCGCGAGCAAGATCCTACACA |  |  |  |
| OT2-For | TACACCCCACCTTGTCCCTCA | GG**A**GGAGGA**A**A**G**CCACGTG**A** GGG | 4:intergenic:TMEM132B-AACS/chr17 | 0.45 |
| OT2-Rev | TGCCTCCCCTCCCCAACAG |  |  |  |
| OT3-For | TACCTCCTGCCTATTTCGCTCT | **A**G**A**GGAG**A**AGAACCACGTG**T** TGG | 4:exon:TENM1/chrX | 0.45 |
| OT3-Rev | CCCAGATGACTTCCGTGTTGA |  |  |  |
| OT4-For | CAGCTCTTGGAATGATGAATTAGG | GGT**A**GAGGAGA**GT**CAC**A**TGG TGG | 4:intergenic:ENSOARG00000004542-TSHZ1/chr23 | 0.42 |
| OT4-Rev | ATGACTGGATACAAAGTGGGAAGA |  |  |  |
| **oOTOF-Exon 6** |  |  |  |  |
| **Primer Name** | **Primer Sequence** | **On-target sequence** | **Number of mismatches:Location/Chromosome** | **CFD Off-target score** |
| OTOF-Ex6-A755 | GCTGAGTCTGTGCTTGTCAGT | GGGGGAGGAGTCCCTTCACG AGG | 0:exon:OTOF/chr3 | 0 |
| OTOF-Ex6-A756 | GAAACCCCTTCCCTCCTCTTG |  |  |  |
|  |  | **Potential off-target sequence** |  |  |
| OT1-For | ACTCTCTTGCCCAAAGGTGAT | GGG**A**GA**AA**AGTCCCTTCAC**A** GGG | 4:intergenic:U6-BMP2K/chr6 | 0.84 |
| OT1-Rev | GTTTCCTGAGGTCCCGGCTTC |  |  |  |
| OT2-For | CTGCCCTATTCCTGCGTGG | GGGGGA**A**GAGTC**T**C**A**TCAC**A** GGG | 4:intergenic:ENSOARG000000190048-COL6A3/chr1 | 0.38 |
| OT2-Rev | ACGAAGGGAGGTGCAGAAATA |  |  |  |
| OT3-For | ACTAGATGGTAAAACAGATGTGGAG | **TTT**GGAG**A**AGTCCCTTCACG TGG | 4:intergenic:5S_rRNA-CPEB2/chr6 | 0.32 |
| OT3-Rev | TCACATGATCTGACCTCGACTTT |  |  |  |
| OT4-For | CACCACACCCTCCTAACCACT | G**A**GG**CG**GG**G**GTCCCTTCACG TGG | 4:intergenic:IL1R1-SNORD86/chr3 | 0.32 |
| OT4-Rev | AAGGACACAGGGCTTACAGA |  |  |  |

CFD: Cutting frequency determination (the closer CFD of 1 the highest possibility of being an off-target site as described by^21^.

Mismatches are in bold.


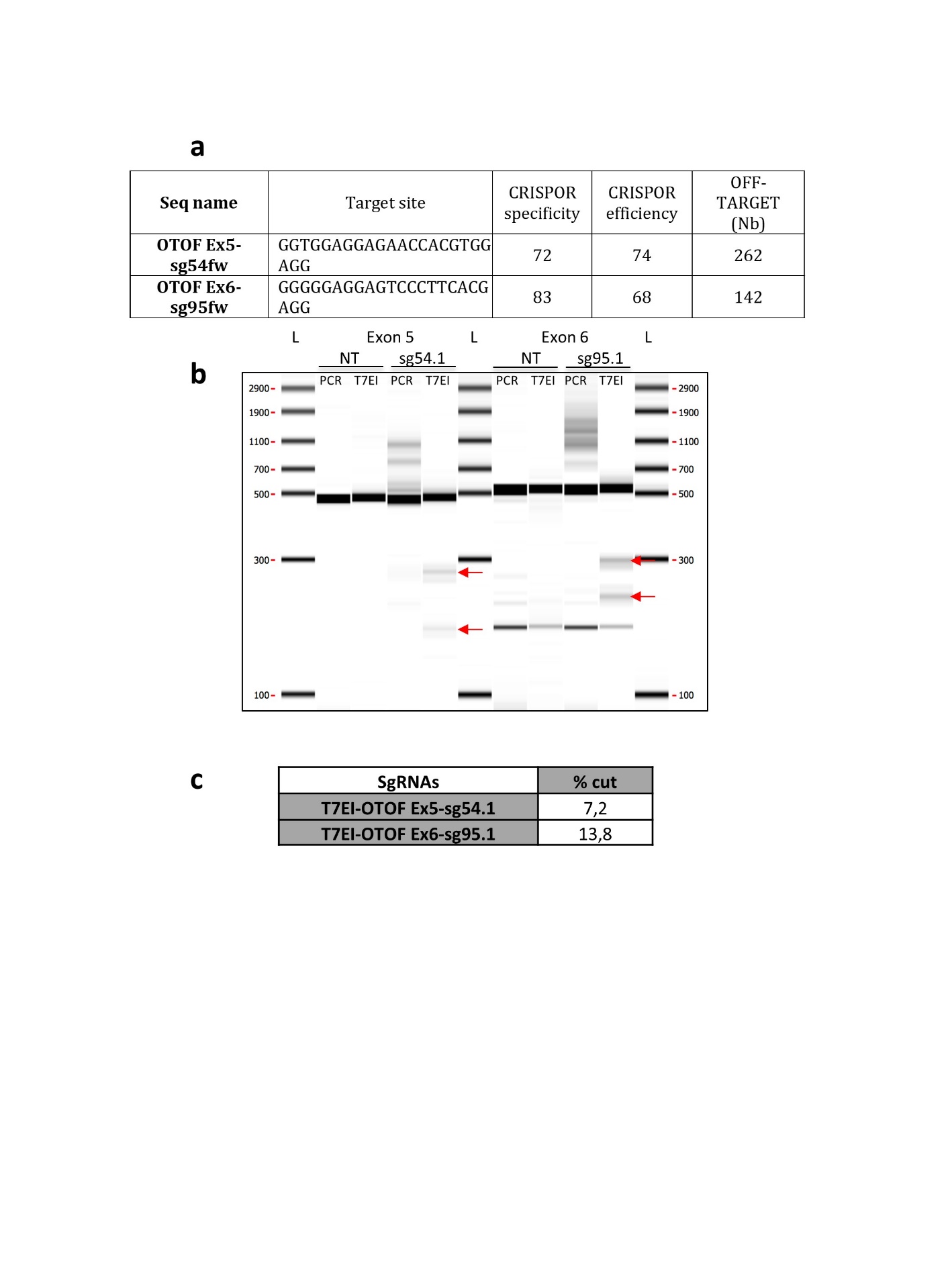


Suppl. Figure S1: in vitro sgRNA validation. **Panel a)** On oOTOF exon 5 and 6, sgRNA were designed via CRISPOR software and chosen for good specificity and efficiency *in silico*. **Panel b)** Each sgRNA was tested *in vitro* by electroporation in sheep cells (A15 cells). L: ladder. NT: non transfected. PCR: amplification of gDNA from A15 cells. T7EI: PCR after T7 Endonuclease I test. Red arrow: bands resulting from T7EI heteroduplexes cleavage. **Panel c)** Efficiencies were assessed after a T7 endonuclease I test followed by a capillary electrophoresis and quantification were done using peak table values.

Supplementary Table S3. Primers list and KI ssODN used for *OTOF* mutation in sheep.

| Primers | Sequence | nt |
| --- | --- | --- |
| OTOF-Ex5-A753 | CACACAGTTGTTGGGGGTCA | 20 |
| OTOF-Ex5-A754 | CTCCTCCCACCGTACCTTTC | 20 |
| OTOF-Ex5-Up2 | GCCACCTTCTCCAACCCCAG | 20 |
| OTOF-Ex5-Lo2 | AGACCCAGCCTAGACCGTG | 19 |
| OTOF-Ex6-A755 | GCTGAGTCTGTGCTTGTCAGT | 21 |
| OTOF-Ex6-A756 | GAAACCCCTTCCCTCCTCTTG | 21 |
| OTOF-Ex6-Up2 | CCCCAGCTCGCCCTCCTTC | 19 |
| OTOF-Ex6-Lo2 | CCAGGCCCCAGGCACACTC | 19 |
| oOTOF-Ex5-4KbUp | GAGACTGAGTGAGCCCAAGT | 20 |
| oOTOF-Ex6-4KbLo | CTCCAAACTCCTCCCCATCCT | 21 |
| oOTOF-Ex6-45KbLo | GCCTGACTCTCCATCAATCCCT | 22 |
| oOTOF-Ex6-6KbLo | ACCCGCTGTTGTTATTGACCC | 21 |
| oOTOF-KOKI | ctgattgggaccttccgcatggtgctgcagaaggtggtggaggagaaccacTAGTGAgtggaggtgaccgacacgctgatggatgacaacaatgcgatcatcaaggtg | 108 |
